# Effective Suppression of HIV-1 Replication by Cytotoxic T Lymphocytes Specific for Pol Epitopes in Conserved Mosaic Vaccine Immunogens

**DOI:** 10.1128/JVI.02142-18

**Published:** 2019-03-21

**Authors:** Chengcheng Zou, Hayato Murakoshi, Nozomi Kuse, Tomohiro Akahoshi, Takayuki Chikata, Hiroyuki Gatanaga, Shinichi Oka, Tomáš Hanke, Masafumi Takiguchi

**Affiliations:** a Center for AIDS Research, Kumamoto University, Kumamoto, Japan; b International Research Center of Medical Sciences, Kumamoto University, Kumamoto, Japan; c AIDS Clinical Center, National Center for Global Health and Medicine, Tokyo, Japan; d The Jenner Institute, University of Oxford, Oxford, United Kingdom; e Nuffield Department of Medicine, University of Oxford, Oxford, United Kingdom; Emory University

**Keywords:** CTL, HIV-1, Pol, conserved epitope, vaccine

## Abstract

Cytotoxic T lymphocytes (CTLs) with strong abilities to suppress HIV-1 replication and recognize circulating HIV-1 could be key for both HIV-1 cure and prophylaxis. We recently designed conserved mosaic T-cell vaccine immunogens (tHIVconsvX) composed of 6 Gag and Pol regions. Since the tHIVconsvX vaccine targets conserved regions common to most global HIV-1 variants and employs a bivalent mosaic design, it is expected that it could be universal if the vaccine works. Although we recently demonstrated that CTLs specific for 5 Gag epitopes in the vaccine immunogens had strong ability to suppress HIV-1 replication *in vitro* and *in vivo*, it remains unknown whether the Pol region-specific CTLs are equally efficient. In this study, we investigated CTLs specific for Pol epitopes in the immunogens in treatment-naive Japanese patients infected with HIV-1 clade B. Overall, we mapped 20 reported and 5 novel Pol conserved epitopes in tHIVconsvX. Responses to 6 Pol epitopes were significantly associated with good clinical outcome, suggesting that CTLs specific for these 6 Pol epitopes had a strong ability to suppress HIV-1 replication in HIV-1-infected individuals. *In vitro* T-cell analyses further confirmed that the Pol-specific CTLs could effectively suppress HIV-1 replication. The present study thus demonstrated that the Pol regions of the vaccine contained protective epitopes. T-cell responses to the previous 5 Gag and present 6 Pol protective epitopes together also showed a strong correlation with better clinical outcome. These findings support the testing of the conserved mosaic vaccine in HIV-1 cure and prevention in humans.

**IMPORTANCE** It is likely necessary for an effective AIDS vaccine to elicit CD8^+^ T cells with the ability to recognize circulating HIV-1 and suppress its replication. We recently developed novel bivalent mosaic T-cell vaccine immunogens composed of conserved regions of the Gag and Pol proteins matched to at least 80% globally circulating HIV-1 isolates. Nevertheless, it remains to be proven if vaccination with these immunogens can elicit T cells with the ability to suppress HIV-1 replication. It is well known that Gag-specific T cells can suppress HIV-1 replication more effectively than T cells specific for epitopes in other proteins. We recently identified 5 protective Gag epitopes in the vaccine immunogens. In this study, we identified T cells specific for 6 Pol epitopes present in the immunogens with strong abilities to suppress HIV-1 *in vivo* and *in vitro*. This study further encourages clinical testing of the conserved mosaic T-cell vaccine in HIV-1 prevention and cure.

## Abstract (precis)

It is likely necessary for an effective AIDS vaccine to elicit CD8^+^ T cells with the ability to recognize circulating HIV-1 and suppress its replication. We recently developed novel bivalent mosaic T-cell vaccine immunogens composed of conserved regions of the Gag and Pol proteins matched to at least 80% globally circulating HIV-1 isolates. Nevertheless, it remains to be proven if vaccination with these immunogens can elicit T cells with the ability to suppress HIV-1 replication. It is well known that Gag-specific T cells can suppress HIV-1 replication more effectively than T cells specific for epitopes in other proteins. We recently identified 5 protective Gag epitopes in the vaccine immunogens. In this study, we identified T cells specific for 6 Pol epitopes present in the immunogens with strong abilities to suppress HIV-1 *in vivo* and *in vitro*. This study further encourages clinical testing of the conserved mosaic T-cell vaccine in HIV-1 prevention and cure.

## INTRODUCTION

HIV-1-specific cytotoxic T lymphocytes (CTLs) play an important role in suppression of HIV-1 replication (1–8). Gag-specific CD8^+^ T-cell responses were the most dominant among the nine proteins of HIV-1 in HIV-1-infected individuals (9). Studies of HIV-1-specific CTLs in chronic HIV-1 infection indeed showed associations of Gag-specific T-cell responses with good clinical outcomes (8, 10–13), indicating that Gag-specific T cells have a stronger ability to suppress HIV-1 replication than responses specific for other proteins. This is explained by relative abundance of Gag protein and reduced viral fitness by escape mutations from Gag-specific CTLs (14–17). On the other hand, a role for Pol-specific CD8^+^ T cells in HIV-1 infection has been only partially analyzed. T-cell responses to Gag and Pol were frequently detected in natural HIV-1 infections (10, 12, 18–20). A study in an HIV-1 clade C-infected African cohort showed that Pol-specific T-cell responses were not associated with a significant reduction in plasma viral load (pVL), whereas Gag-specific responses significantly correlated with lower pVL (10). In contrast, a study of clade B-infected treatment-naive Japanese individuals demonstrated that in addition to Gag-specific T cells, Pol-specific T cells also had a strong ability to suppress HIV-1 replication *in vivo* (20–22).

Although great efforts in T-cell vaccine development have been invested, no clinical trial has shown a definitive effect regarding prevention of HIV-1 infection (23, 24). This is because the vaccine-elicited T cells may fail to recognize escape mutant viruses and/or the vaccines may fail to elicit strong T-cell immunity and suppress HIV-1 replication. To minimize escape and target HIV-1 “where it hurts,” vaccines using conserved regions of HIV-1 proteins as immunogens have been proposed (25–28). Ondondo et al. recently designed a second-generation conserved-region T-cell mosaic vaccine, tHIVconsvX, which consists of 2 Gag and 4 Pol protein regions functionally conserved across all M group viruses with high coverage of known protective epitopes and employs a bioinformatically designed bivalent mosaic to maximize the match of the vaccine potential T-cell epitopes to the global circulating HIV-1 isolates (29). Initial study of T cells recognizing the tHIVconsvX immunogens showed a significant correlation of both the total magnitude and breadth of the tHIVconsvX immunogen-specific T-cell responses to lower pVLs and higher CD4^+^ T-cell counts (CD4 counts) in 120 treatment-naive HIV-1 clade B-infected patients in Japan (29). A following study demonstrated that CD8^+^ T cells specific for five Gag epitopes in tHIVconsvX immunogens contribute to suppression of HIV-1 replication *in vivo* (30). However, it remains unknown whether CD8^+^ T cells specific for the Pol region in the immunogen are equally effective.

In the present study, we clarified the role of CD8^+^ T cells specific for the Pol regions in the tHIVconsvX immunogens in 200 HIV-1-infected Japanese individuals. We determined the fine specificities and HLA restriction of CD8^+^ T cells specific for the Pol regions in the immunogens and further analyzed the correlation of these Pol epitope-specific T cells to clinical outcome as well as assessed their HIV-1 inhibition capacity *in vitro*. These results will inform and encourage clinical testing of the second-generation conserved-mosaic T-cell vaccines.

## RESULTS

### CD8^+^ T-cell responses to Pol peptides derived from the tHIVconsvX immunogens.

**FIG 1:**
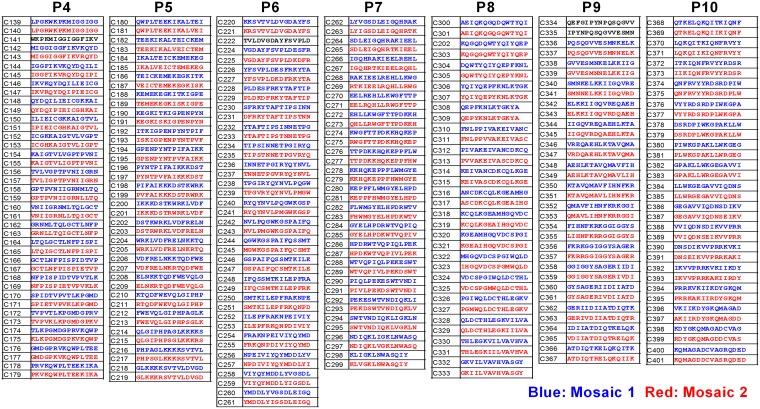
Overlapping peptide pairs (15-mer) in Pol peptide pools. Seven Pol pools, P4 to P10, contain pairs of 15-mer Pol peptides overlapped by 11 amino acids covering two mosaic regions in the tHIVconsvX immunogen (29). Each pool contains 17 to 21 pairs of the 15-mer peptides. P4, P5, P6, P7, P8, P9, and P10 cover Pol amino acids 94 to 188, 178 to 268, 258 to 352, 342 to 426, 482 to 510/741 to 798, 852 to 934, and 924 to 1003, respectively.

**FIG 2:**
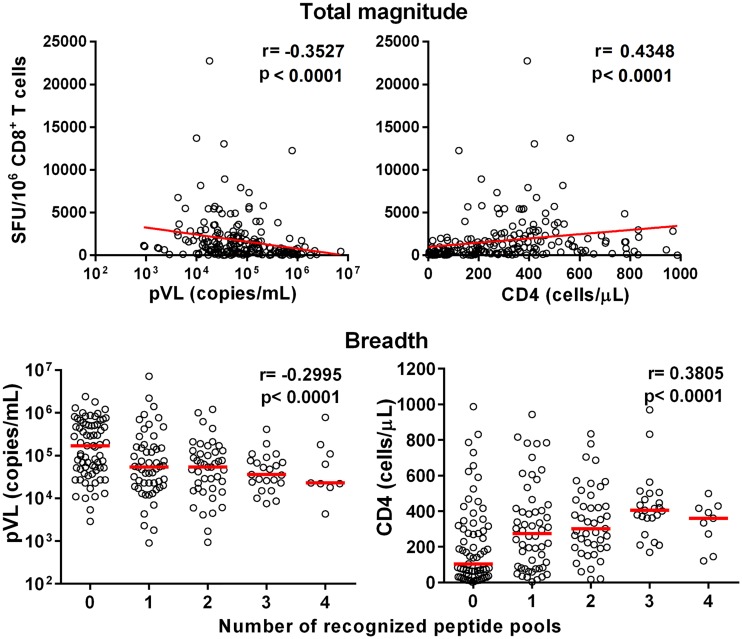
Correlation of total magnitude and breadth of T-cell responses to 7 Pol pools with pVL and CD4 count. T-cell responses to the 7 Pol peptide pools derived from the tHIVconsvX immunogen were analyzed using an IFN-γ ELISPOT assay in 200 treatment-naive HIV-1-infected Japanese individuals. Correlation coefficients (*r*) and *P* values were determined by using the Spearman rank correlation test.

**FIG 3:**
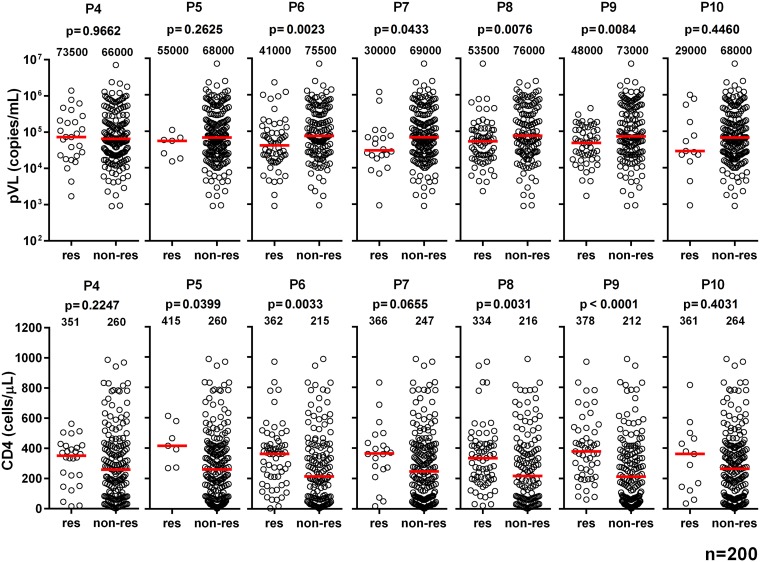
Association of T-cell responses to each Pol peptide pool with pVL and CD4 count. T-cell responses to each Pol peptide pool were determined by IFN-γ ELISPOT assay in 200 treatment-naive HIV-1-infected Japanese individuals. We statistically analyzed differences in pVL and CD4 count between responders (res) and nonresponders (non-res) using the Mann-Whitney test. The value in each graph represents the median of pVL and CD4 count.

We generated 15-mer Pol peptides overlapped by 11 amino acids covering the two mosaic regions for Pol proteins in the tHIVconsvX immunogens (Fig. 1). First, we measured the T-cell responses to Pol peptide pools (P4 to P10) in 200 HIV-1-infected treatment-naive Japanese individuals using a gamma interferon (IFN-γ) enzyme-linked immunosorbent spot (ELISPOT) assay, followed by analyses of the correlation between the total magnitude or breadth of CD8^+^ T cells specific for Pol peptide pools and pVL or CD4 count. The total magnitude and breadth of these responses were significantly correlated inversely with pVL and positively with CD4 count in these individuals (Fig. 2). These results suggested that CD8^+^ T cells specific for Pol epitopes in the immunogens suppress HIV-1 replication in this cohort. To further detail the association between CD8^+^ T cell responses to each peptide pool and clinical outcome, we compared pVL and CD4 count in each pool between responders and nonresponders. The responders to P6, P8, and P9 peptides showed both significantly lower pVL and higher CD4 count than nonresponders (Fig. 3), suggesting a protective role against HIV-1 infection *in vivo*.

### Mapping of the CD8^+^ T-cell specificity to optimal Pol epitopes in the tHIVconsvX immunogens.

**FIG 4:**
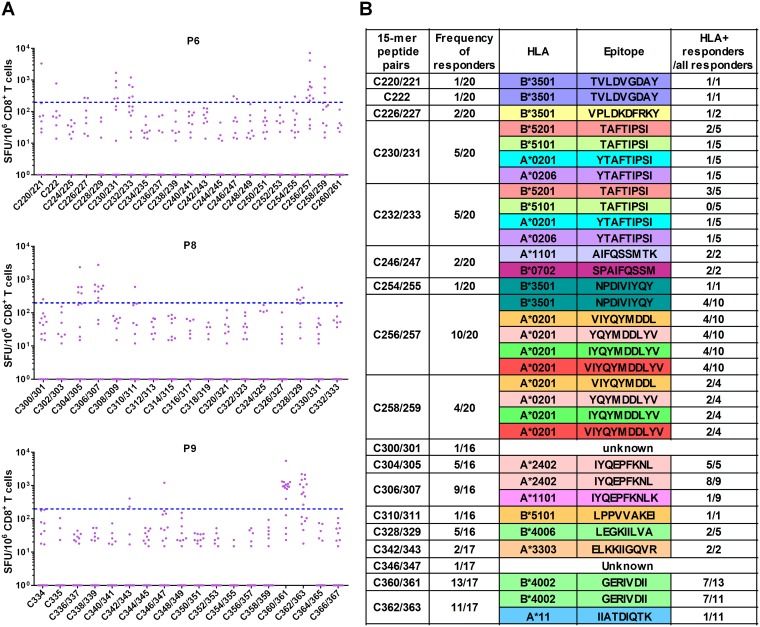
T-cell responses to 15-mer peptide pairs in Pol peptide pools. (A) T-cell responses to 15-mer peptide pairs in P6, P8, and P9. The responses to the 15-mer peptide pairs were analyzed by IFN-γ ELISPOT assay in 20 responders to P6, 16 responders to P8, and 17 responders to P9. The dotted line at 200 SFU/10^6^ CD8^+^ T cells indicates a threshold for a positive response. (B) Summary of responders and peptide pairs. Reported epitopes in the 15-mer peptide pairs of P6, P8, and P9 are shown according to the Los Alamos National Laboratory HIV Sequence Database as of November 2018. HLA^+^ responders are those with the matching restricting HLA allele for the reported epitope.

**FIG 5:**
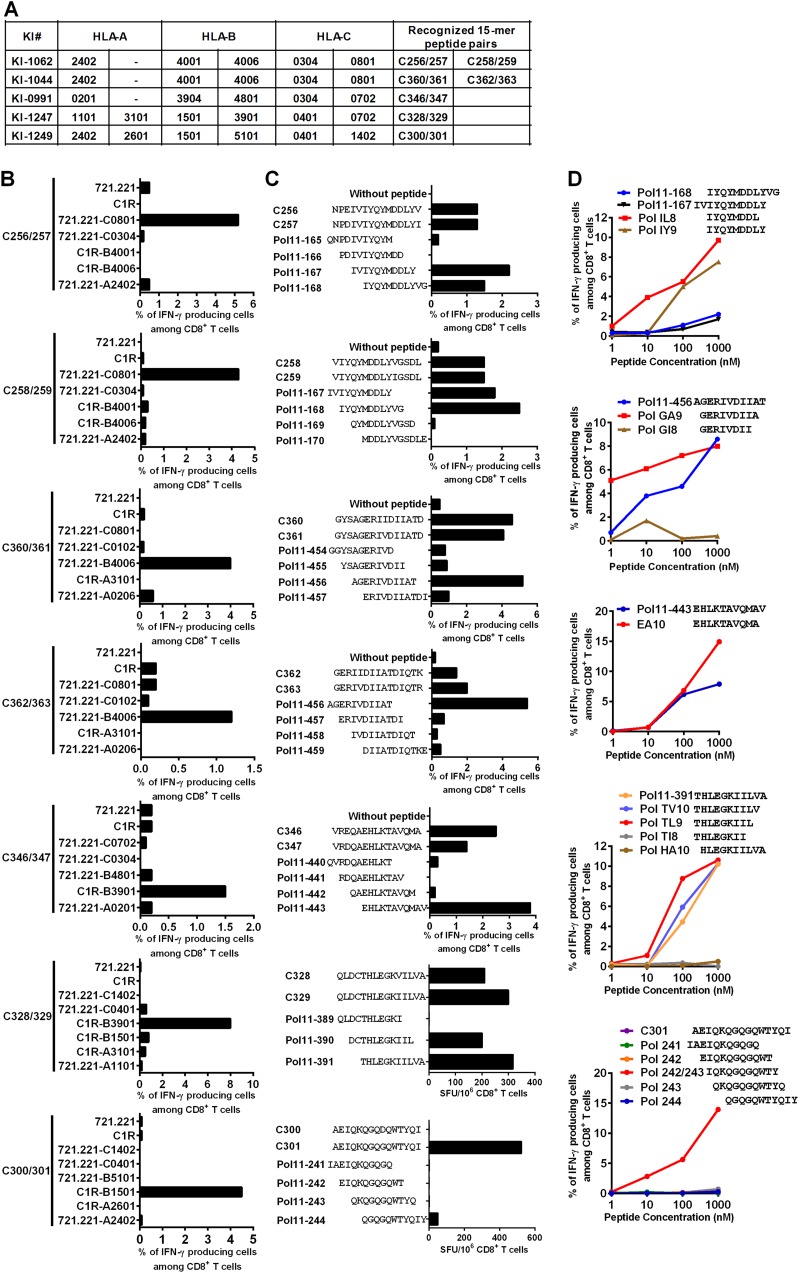
Identification of novel Pol epitopes in tHIVconsvX. (A) Peptide pairs recognized by T cells in 5 individuals KI-1062, KI-1044, KI-991, KI-1247, and KI-1249, who did not have matching HLA alleles for previously reported epitopes. (B) HLA restrictions of T-cell responses to 15-mer overlapping peptides. T-cell responses of STCL stimulated with C1R or 721.221 cells expressing individual HLA molecule shared by the responders and pulsed with the peptide pair were analyzed by ICS assay. C256/257 and C258/259, C360/361 and C362/363, C346/347, C328/329, and C300/301 peptide pairs were analyzed by using STCLs derived from KI-1062, KI-1044, KI-0991, KI-1247, and KI-1249, respectively. (C) Identification of overlapping 11-mer HIV-1 clade B peptides recognized by the T cells restricted by HLA-C*08:01, HLA-B*40:06, HLA-B*39:01, and HLA-B*15:01. The T-cell responses of STCLs expanded with C256/257, C360/361, and C346/347 peptide pairs to the corresponding stimulator cells prepulsed with overlapping 11-mer HIV-1 clade B-derived peptides covering the 15-mers were analyzed in ICS assay. For C328/329 or C300/301, the T-cell responses to overlapping 11-mer peptides covering each 15-mer peptide pair were analyzed by using IFN-γ ELISPOT assay. (D) Identification of optimal epitope peptides. The STCL responses stimulated with C256/257, C360/361, C346/347, Pol11-391, or C301 to the corresponding stimulator cells pre-pulsed with individual truncated peptides were analyzed by ICS assay.

We sought to map Pol epitopes included in P6, P8, and P9. We selected, respectively, 20, 16, and 17 individuals based on sufficient peripheral blood mononuclear cells (PBMCs) available for the determination of optimal epitopes. We found T-cell responses to 8 peptide pairs and one common single peptide in P6, 5 peptide pairs in P8, and 4 peptide pairs in P9 in at least one individual (Fig. 4A). These 15-mer peptides contained sequences of previously reported epitopes: 13 epitopes in P6, 4 epitopes in P8, and 3 epitopes in P9 (Fig. 4B). Upon inspection of the subjects’ HLA molecules, most of the responders were found to have HLA alleles previously reported to restrict these optimal epitopes. However, all or some responders to 15-mer peptide pairs C256/257, C258/259, C300/301, C328/329, C346/347, C360/361, and C362/363 did not have the matching HLA alleles (Fig. 5A), suggesting that their CD8^+^ T cells may recognize novel, previously unreported epitopes.

We sought to identify these novel epitopes and their restricting HLA molecules. Subjects’ PBMCs were first expanded with each peptide pair for 12 to 14 days, and these short-term cell lines (STCLs) were tested in an intracellular cytokine staining (ICS) assay using either C1R or 721.221 cells transfected with all subjects’ HLA class I molecules. T-cell responses to these peptides were restricted by the following HLA alleles: C256/257 and C258/259 by HLA-C*08:01, C360/361 and C362/363 by HLA-B*40:06, C346/347 and C328/329 by HLA-B*39:01, and C300/301 by HLA-B*15:01 (Fig. 5B).

Next we identified optimal epitopes by using overlapping 11-mer peptides and their truncated ones. Both STCLs specific for C256/257 and C258/259 responded to Pol11-167/168 peptides (Fig. 5C), suggesting that these two STCLs were specific for the same epitope. By using truncated peptides, we demonstrated novel epitope IYQYMDDL (Pol IL8) restricted by HLA-C*08:01 (Fig. 5D). In addition, T cells specific for C360/361 and C362/363 recognized Pol 11-456 peptides, while T cells specific for C346/347 and C328/329 recognized Pol 11-443 and Pol 11-390/391, respectively (Fig. 5C). By using truncated peptides again, we identified novel epitopes GERIVDIIA (Pol GA9) restricted by HLA-B*40:06 and EHLKTAVQMA (Pol EA10) and THLEGKIIL (Pol TL9) restricted by HLA-B*39:01 (Fig. 5D). Although we identified an HLA-B*15:01-restricted epitope in C300/301, none of the 11-mer peptides covering C300/301 were recognized by the C300/301-specific T cells (Fig. 5C). Since HLA-B*15:01-binding peptides have 2 anchor residues, Q at position 2 and Y at the C terminus (31), we speculated that IQKQGQGQWTY (IY11) between Pol11-242 and -243 (Pol232/233) might be the optimal peptide restricted by HLA-B*15:01. Indeed, it was confirmed by ICS assay that IY11 was an optimal epitope (Fig. 5D). Thus, we identified five novel epitopes.

### Pol epitope-specific CD8^+^ T cells have strong abilities to suppress HIV-1 replication *in vivo*.

As shown above, we mapped 20 reported and 5 novel HIV-1 Pol epitopes in HIV-1-infected Japanese individuals who responded to tHIVconsvX immunogen-derived Pol peptides. To further investigate whether T cells specific for these epitopes have ability to inhibit HIV-1 replication *in vivo*, we selected 221 individuals whose PBMCs were available for this analysis and analyzed their T-cell responses to these epitope peptides in the IFN-γ ELISPOT assay. Since a T-cell response to IK9/HLA-A11 was detected in only one patient (Table 1), we excluded this epitope from further statistical analysis. We analyzed differences in pVL or CD4 count between responders to each epitope and nonresponders. Responders to 10 epitopes (YI9/HLA-A*02:06, TI8/HLA-B*51:01, SM9/HLA-B*07:02, TI8/HLA-B*52:01, IL9/HLA-A*24:02, ER10/HLA-A*33:03, LI9/HLA-B*51:01, LA9/HLA-B*40:06, GI8/HLA-B*40:02, and GA9/HLA-B*40:06) had significantly lower pVLs and/or higher CD4 counts than nonresponders, suggesting that T cells specific for these 10 epitopes have the ability to suppress HIV-1 replication *in vivo* (Table 1).

**TABLE 1:** Association of CTL responses to Pol epitopes with pVL and CD4 count in 221 HIV-1-infected Japanese individuals

Epitope	Sequence	HLA	Frequency		Median pVL		Median CD4 count		*P* value^*b*^	
			Res	Non-res	Res	Non-res	Res	Non-res	pVL	CD4
TY9	TVLDVGDAY	B*35:01	8	213	78,500	58,000	229	304	0.2884	0.4107
VY10	VPLDKDFRKY	B*35:01	9	212	49,000	63,500	443	301	0.5938	0.2094
NY9	NPDIVIYQY	B*35:01	12	209	48,000	62,000	408	294	0.3128	0.0613
YI9	YTAFTIPSI	A*02:01	4	217	123,500	58,000	497	300	0.4933	0.2646
VL9	VIYQYMDDL	A*02:01	6	215	89,000	58,000	240	301	0.4458	0.4345
YV9	YQYMDDLYV	A*02:01	7	214	28,000	63,500	281	303	0.4092	0.9894
IV10	IYQYMDDLYV	A*02:01	11	210	69,000	59,500	281	303	0.9762	0.6981
VV11	VIYQYMDDLYV	A*02:01	13	208	69,000	59,500	281	303	0.9006	0.6055
IK9	IIATDIQTK	A*11	1	220						
AK9	AIFQSSMTK	A*11:01	4	217	51,500	61,000	377	300	0.7077	0.4529
IK10	IYQEPFKNLK	A*11:01	3	218	70,000	59,500	392	301	0.8456	0.8758
YI9	YTAFTIPSI	A*02:06	6	215	20,500	65,000	431	294	**0.0433**	**0.0352**
SM9	SPAIFQSSM	B*07:02	4	217	14,500	65,000	470	300	**0.0151**	0.1277
TI8	TAFTIPSI	B*52:01	10	211	25,000	68,000	513	294	**0.0062**	**0.0061**
TI8	TAFTIPSI	B*51:01	11	210	18,000	68,000	408	293	**0.0002**	0.1279
LI9	LPPVVAKEI	B*51:01	31	190	29,000	68,000	389	285	**0.0201**	**0.0115**
IL9	IYQEPFKNL	A*24:02	17	204	23,000	68,500	416	297	**0.001**	**0.042**
ER10	ELKKIIGQVR	A*33:03	14	207	23,500	67,500	427	294	**0.012**	0.1109
GI8	GERIVDII	B*40:02	12	209	27,000	68,000	390	294	**0.0271**	**0.0204**
LA9	LEGKIILVA	B*40:06	19	202	38,000	67,500	366	290	**0.0497**	**0.018**
GA9^*a*^	GERIVDIIA	B*40:06	21	200	23,000	68,500	437	284	**0.0014**	**0.0002**
IL8^*a*^	IYQYMDDL	C*08:01	15	206	36,000	66,000	366	293	0.0642	0.0609
TL9^*a*^	THLEGKIIL	B*39:01	8	213	62,000	61,000	328	300	0.946	0.8956
EA10^*a*^	EHLKTAVQMA	B*39:01	5	216	54,000	61,500	334	301	0.9358	0.914
IY11^*a*^	IQKQGQGQWTY	B*15:01	8	213	54,500	62,000	339	300	0.865	0.9967

a New epitope.

b Statistically analyzed differences in pVL or CD4 count between responders (res) and nonresponders (non-res) by Mann-Whitney test. Bold indicates that differences were statistically significant.

We further analyzed the association of responses to these 10 epitopes with pVL and CD4 count in individuals having the epitopes’ restricting HLA molecules. We found that responders to 6 epitopes (TI8/HLA-B*52:01, LI9/HLA-B*51:01, IL9/HLA-A*24:02, ER10/HLA-A*33:03, GI8/HLA-B*40:02, and GA9/HLA-B*40:06) had significantly lower pVLs and higher CD4 counts than the nonresponders with the same HLA alleles (Table 2). We confirmed a previously reported inhibition of HIV-1 *in vitro* and *in vivo* by T cells specific for the GI8/HLA-B*40:02 epitope (20, 32). Thus, the present study showed that T cells specific for the 5 epitopes TI8, LI9, ER10, IL9, and GA9 could efficiently suppress HIV-1 replication *in vivo*.

**TABLE 2:** Association of CTL responses to Pol epitopes with pVL and CD4 count in the Japanese individuals having HLA alleles restricting each epitope

Epitope	Sequence	HLA	Frequency		Median pVL		Median CD4 count		*P* value^*b*^	
			Res	Non-res	Res	Non-res	Res	Non-res	pVL	CD4
LA9	LEGKIILVA	B*40:06	19	11	38,000	46,000	366	304	0.8905	0.7031
YI9	YTAFTIPSI	A*02:06	6	28	20,500	44,500	431	252	0.2166	0.0429
TI8	TAFTIPSI	B*51:01	11	30	18,000	84,000	408	274	**0.0018**	0.2279
SM9	SPAIFQSSM	B*07:02	4	34	14,500	84,500	470	258	**0.0056**	0.0812
TI8	TAFTIPSI	B*52:01	10	41	25,000	67,000	513	276	**0.0038**	**0.034**
GI8	GERIVDII	B*40:02	12	20	27,000	125,000	390	183	**0.0005**	**0.0263**
IL9	IYQEPFKNL	A*24:02	17	116	23,000	83,000	416	288	**0.0001**	**0.0337**
ER10	ELKKIIGQVR	A*33:03	14	17	23,500	76,000	427	196	**0.0152**	**0.0253**
LI9	LPPVVAKEI	B*51:01	31	10	29,000	125,000	389	110	**0.0151**	**0.0001**
GA9^*a*^	GERIVDIIA	B*40:06	21	9	23,000	72,000	437	259	**0.0436**	**0.0059**

a New epitope.

b Statistically analyzed differences in pVL or CD4 count between responders and nonresponders by Mann-Whitney test. Bold indicates that differences were statistically significant.

### *In vitro* suppression of HIV-1 replication by IL9-, ER10-, and GA9-specific T cells.

**FIG 6:**
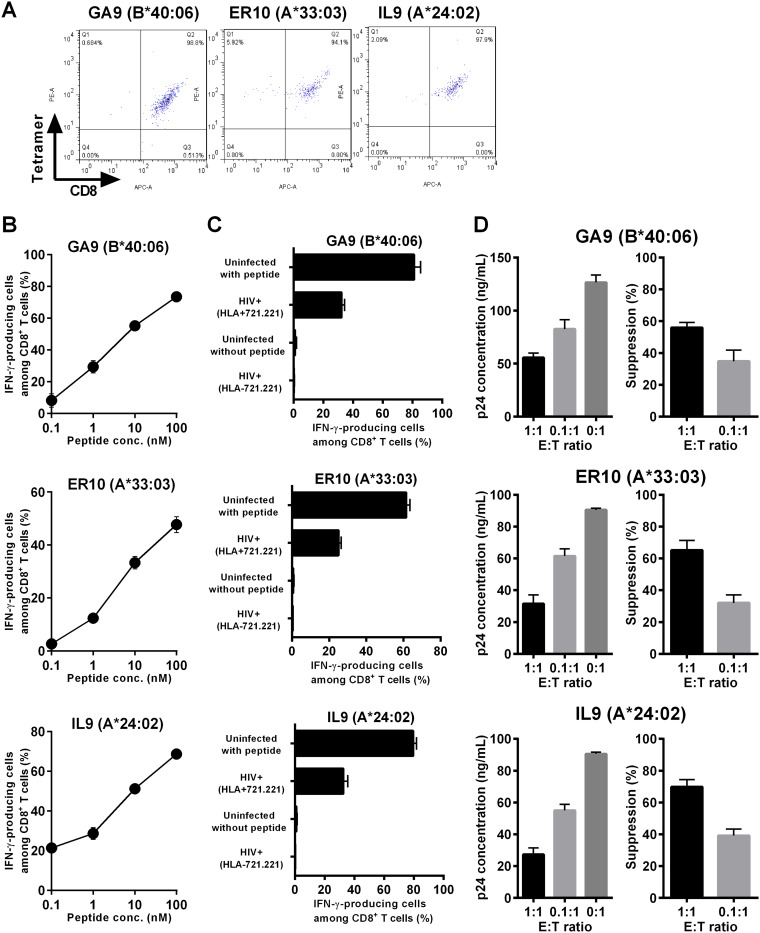
Ability of CTLs to recognize HIV-1-infected cells and to suppress HIV-1 replication *in vitro*. (A) T-cell lines specific for GA9, ER10, or IL9 were established from PBMCs of HLA-B*40:06^+^ KI-1268, HLA-A*33:03^+^ KI-1427, and HLA-A*24:02^+^ KI-1105 individuals as shown in Materials and Methods. These T-cell lines were stained with the specific tetramers. (B) Recognition of GA9, ER10, and IL9 peptides by epitope-specific CD8^+^ T cells. The epitope-specific T-cell lines were stimulated with epitope peptide-pulsed 721.221 cells expressing the corresponding HLA alleles, and then IFN-γ production from these T-cell lines was analyzed by performing the ICS assay. The results are shown as means and SDs (*n* = 3). (C) Recognition of HIV-1-infected cells by T cells specific for the GA9, ER10, or IL9 epitope. The T-cell lines were stimulated with HIV-1 NL4-3-infected 721.221 cells (HIV+) expressing CD4 and the corresponding HLAs or HLA-negative 721.221 cells, and IFN-γ production from the T cells was measured by the ICS assay. The proportions of 721.221-B4006, -A3303, and -A2402 and HLA-negative 721.221 cells infected with HIV-1 NL4-3 were 37.6%, 24.5%, 38.3%, and 37.3%, respectively. (D) Suppression of HIV-1 replication by the T-cell lines specific for GA9, ER10, or IL9. Primary CD4^+^ T cells from healthy donors carrying the corresponding HLA alleles were infected with HIV-1 NL4-3 and then cocultured with epitope-specific T cells at E:T ratios of 1:1 and 0.1:1. The concentration of Gag p24 in the culture supernatant was measured by using an enzyme-linked immunosorbent assay. The percentage of suppression was calculated as follows: (concentration of Gag p24 without CTLs – concentration of Gag p24 with CTLs)/concentration of Gag p24 without CTLs × 100. The data are presented as means and SDs (*n* = 3).

We previously demonstrated a strong virus-suppressive ability of CTLs specific for TI8/HLA-B*52:01 and LI9/HLA-B*51:01 *in vitro* (33, 34). We further investigated the ability of T cells specific for 3 other epitopes—GA9/HLA-B*40:06, ER10/HLA-A*33:03, and IL9/HLA-A*24:02—to suppress HIV-1 replication *in vitro*. We established T-cell lines specific for GA9, ER10, and IL9 from PBMCs of HLA-B*40:06^+^ (KI-1268), HLA-A*33:03^+^ (KI-1427), and HLA-A*24:02^+^ (KI-1105) individuals, respectively, after fluoreccence-activated cell sorting (FACS) using HLA/peptide tetrameric complexes (Fig. 6A). We analyzed recognition by these T-cell lines of peptide-pulsed and HIV-1-infected target cells using the ICS assay. All of the three T-cell lines responded to the specific peptides even at the low peptide concentration of 1 nM (Fig. 6B) and efficiently recognized HIV-1-infected target 721.221 cells expressing CD4 and the corresponding HLAs, but not uninfected or HLA-untransfected 721.221 cells infected with HIV-1 (Fig. 6C). Finally, we evaluated the ability of these T cells to suppress HIV-1 replication by viral-suppression assay. The results showed that they efficiently suppressed HIV-1 replication *in vitro* (Fig. 6D).

### Cross-recognition of IL9 and GA9 variants by specific T cells.

**FIG 7:**
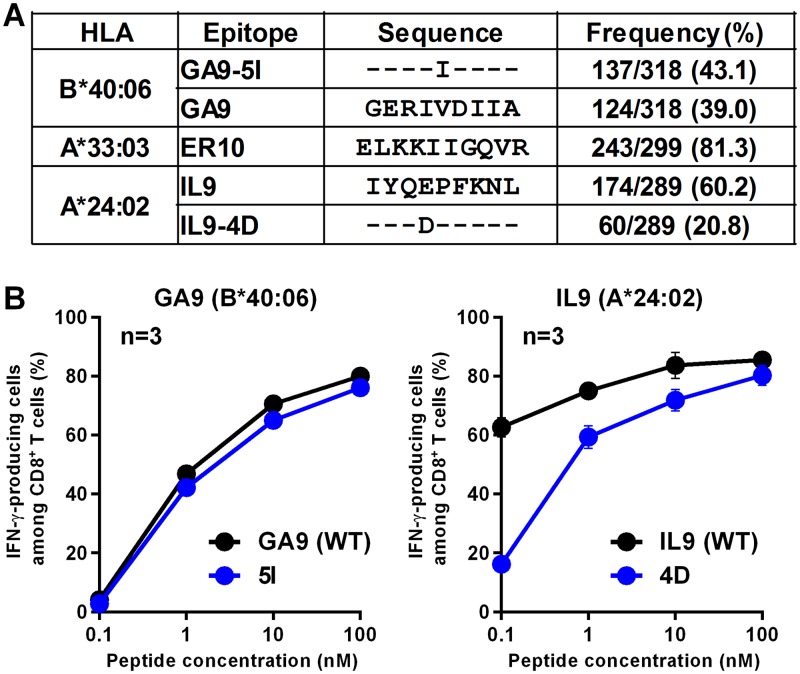
Recognition of variant epitope peptides by T cells specific for IL9 and GA9. (A) Frequencies of IL9, ER10, and GA9 mutant epitopes in Japanese individuals. The frequencies of mutant epitopes were investigated in chronically HIV-1-infected Japanese individuals. (B) The responses of the T-cell lines specific for IL9 or GA9 to wild-type or mutant peptide-prepulsed 721.221 cells expressing the corresponding HLA alleles were analyzed by using the ICS assay. The results are shown as means and SDs (*n* = 3).

We analyzed the sequences of the IL9-, ER10-, and GA9 Pol epitopes in our cohort of Japanese patients and found variations in IL9 and GA9 (Fig. 7A), while the sequence of ER10 was conserved in more than 80% of the individuals. We therefore assessed cross-recognition of variant epitopes IL9-4D and GA9-5I by IL9- and GA9-specific CD8^+^ T cells, respectively. We found cross-recognition in both cases (Fig. 7B). Thus, T cells specific for IL9, GA9, and ER10 can recognize more than 80% of circulating HIV-1 in Japan.

### Impact of T cells specific for Gag and Pol epitopes in tHIVconsvX immunogens on suppression of HIV-1 replication *in vivo*.

**FIG 8:**
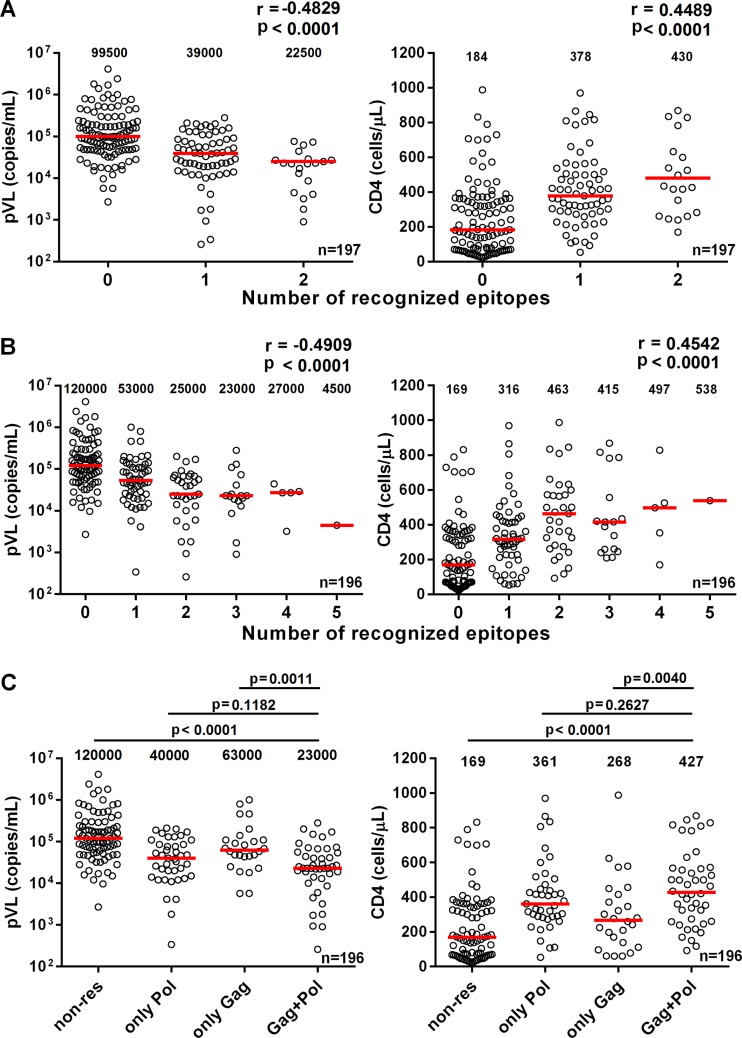
Correlation of T-cell responses to protective Gag and Pol epitopes with clinical parameters in HIV-1-infected individuals. T-cell responses to 5 Gag (RI8, WV8, AA9, HR10, and TL8) and 6 Pol (TI8, IL9, LI9, GA9, GI8, and ER10) epitope peptides were analyzed by using an IFN-γ ELISPOT assay. The correlation of T-cell responses to protective 6 Pol epitopes (A) or 11 protective Gag and the Pol epitopes (B) with pVL and CD4 count was statistically analyzed using Spearman rank test. The pVL and CD4 count in responders to both Pol and Gag epitopes, to only Gag epitopes, and to only Pol epitopes as well as nonresponders were statistically analyzed by Mann-Whitney test (C). The value in each graph represents the median pVL and CD4 count. The averages of numbers of epitopes recognized by T cells are 1.17, 1.23, and 2.66 in only Pol, only Gag, and Gag and Pol groups, respectively. The *P* value between responders to only Pol epitopes and nonresponders was <0.0001 (pVL and CD4 count), while those between responders to only Gag epitopes and nonresponders was 0.032 (pVL) and 0.055 (CD4 count).

To investigate the impact of the T cells specific for the 6 protective Pol epitopes IL9/HLA-A*24:02, ER10/HLA-A*33:03, GI8/HLA-B*40:02, GA9/HLA-B*40:06, LI9/HLA-B*51:01, and TI8/HLA-B*52:01 on suppression of HIV-1 replication *in vivo*, we analyzed correlations between the breadth of T-cell responses to the 6 epitopes and clinical parameters in the Japanese individuals. The breadth of the responses to the epitopes was correlated inversely with pVL and positively with CD4 count (Fig. 8A), suggesting that these Pol epitope-specific T cells play an important role in suppression of HIV-1 replication in this cohort. We recently demonstrated that the T cells specific for 5 Gag epitopes HR10/HLA-A*33:03, TL8/HLA-B*40:02, RI8/HLA-B*52:01, WV8/HLA-B*52:01, and AA9/HLA-A*02:06 in the tHIVconsvX immunogens had a strong ability to control HIV-1 *in vivo* (30). In order to predict an effect of T cells specific for these all protective epitopes on control of HIV-1, we analyzed correlations between T-cell responses to these 11 epitopes and clinical parameters. The breadth of the responses to the 11 epitopes showed highly significant correlations with both lower pVLs (*P* < 1 × 10^−4^; *r* = −0.4909) and higher CD4 counts (*P* < 1 × 10^−4^; *r* = 0.4542) in these Japanese individuals (Fig. 8B). Responders to both Pol and Gag epitopes had significantly lower pVLs and higher CD4 counts than responders to only Gag epitopes, though they had only a trend of lower pVLs and higher CD4 counts than with responders to only Pol epitopes (Fig. 8C). These results suggest additional protective effects of Gag-specific T-cell responses with Pol-specific ones on suppression of HIV-1 replication. Thus, the results indicate that all the strongly protective epitopes we have identified in the tHIVconsvX immunogens play an important role in immunity against HIV-1 clade B infection in Japan.

## DISCUSSION

Previous cross-sectional analysis of T-cell responses to 18-mer overlapping HIV-1 peptides spanning the entire HIV-1 proteome in approximately 600 clade C-infected Africans demonstrated that a breadth of T-cell responses to Gag peptides was inversely associated with pVL, but those to peptides in other proteins were not (10). In addition, analyses of T-cell responses in clade B-infected individuals at a small population level showed inverse correlation of Gag-specific T-cell responses with pVL but not to any other HIV-1 protein (12, 35). Thus, it is well documented that Gag-specific T cells play a critical role in controlling HIV-1 in infected individuals. On the other hand, there is growing evidence that Pol-specific CD8^+^ T cells have the ability to suppress HIV-1 replication. A previous study using a conserved immunogen showed that CD8^+^ T cells specific for Pol peptides have a stronger ability to suppress HIV-1 replication *in vitro* than those specific for Gag, Env, and Vif peptides in healthy volunteers vaccinated with the first-generation conserved immunogen HIVconsv employing alternating clade consensus sequences (26). Moreover, we showed that both T-cell responses to Gag and Pol were significantly correlated with lower pVLs and higher CD4 counts in antiretroviral therapy (ART)-free HIV-1 clade B-infected Japanese individuals (20). In the present study, we demonstrated that six Pol epitope-specific CD8^+^ T cells had strong associations with both lower pVLs and higher CD4 counts in treatment-naive HIV-1-infected Japanese individuals carrying HLA alleles restricting each epitope, suggesting that the 6 Pol epitope-specific T cells contribute to suppression of HIV-1 *in vivo*. In addition, we confirmed effective suppression of HIV-1 replication by T cells specific for the Pol ER10, IL9, and GA9 epitopes *in vitro*, while previous studies demonstrated that CTLs specific for TI8, LI9, and GI8 had strong abilities to suppress HIV-1 *in vitro* (32–34). Our recent study showed that T cells specific for five Gag epitopes in this vaccine immunogens effectively suppress HIV-1 replication (30). These findings together suggested that CD8^+^ T cells specific for the six Pol and the five Gag epitopes can inhibit HIV-1 replication. It is our working hypothesis that when studying correlates of T-cell protection, the T-cell specificity is critically important. Any attempts to identify T-cell correlates using the full-length proteins is likely to yield a blurred picture. Much more granularity is required, and a clearer correlation can be achieved associating T-cell protection with responses to the common functionally conserved regions and/or protective epitopes.

The six protective Pol epitopes identified in the present study are restricted by the HLA-A*24:02, HLA-A*33:03, HLA-B*40:02, HLA-B*40:06, HLA-B*51:01, and HLA-B*52:01 alleles. In our cohort, 81% (1,976/2,443 [data not shown]) of Japanese individuals have at least one of these six alleles. In Caucasians, only HLA-A*24:02 (17%) and HLA-B*51:01 (10%) are also frequent (36), and the six alleles are rarely found in Africans (37–39). Low frequencies of these HLA alleles in Caucasians and Africans may account for the fact that the protective Pol epitopes were not previously identified in them. Similar studies defining protective epitopes and their restriction HLA alleles in Caucasians and Africans are ongoing, although availability of treatment-naive HIV-1-positive cohorts is rare.

The usefulness of these epitopes may extend to the Chinese population, for whom the restricting alleles also have a good representation of HLA-A*24:02 (30%), HLA-A*33:03 (22%), HLA-B*51:01 (9%), HLA-B*52:01 (8%), and HLA-B*40:06 (7%) (40). Analysis of T-cell responses to HLA-B*51:01-restricted epitopes in 22 ART-free HIV-1 clade B-infected Chinese individuals demonstrated that the PolLI9-specific T cells were present in approximately 80% of the individuals (41), which is consistent with the present study in Japanese individuals (76%). Although the above-mentioned study did not analyze association of the T-cell responses with clinical outcome due to the low number of patients tested (41), we here clearly establish that the LI9-specific T-cell response was significantly associated with good clinical outcome in the Japanese individuals. In addition, our previous study demonstrated that T-cell clones specific for LI9 effectively suppress HIV-1 replication *in vitro* (33).

In Japan, the ER10 epitope was conserved among circulating viruses, whereas variations were found within IL9, GA9, LI9, GI8, and TI8. We here demonstrate that T-cell lines specific for IL-9 and GA9 cross-recognized the variant peptides IL9-4D and GA9-5I, respectively. In addition, previous studies showed that T-cell clones specific for GI8 and LI9 evenly recognized their mutant peptides (32, 41), whereas TI8-specific HLA-B*52:01-restricted T cells failed to recognize peptides mutated at the C terminus (34). We show here that the TI8-specific T cells had the ability to suppress HIV-1 replication *in vivo* (Table 2), suggesting that the ability of the T cells to suppress replication of wild-type virus may contribute to the suppression of HIV-1 replication at a population level.

In summary, we demonstrated strong abilities of the T cells specific for six conserved protective Pol epitopes present in the tHIVconsvX immunogens to control HIV-1 *in vivo*. Together with the five Gag epitopes identified in our previous study, broadly specific T-cell responses to 11 protective epitopes (5 in Gag and 6 in Pol) correlated strongly with low pVLs and high CD4 counts in HIV-1 clade B-infected Japanese individuals. Therefore, these findings indicate that the second-generation conserved mosaic tHIVconsvX immunogens contain a number of very useful protective epitopes. If these immunogens can induce high frequencies of these T cells in the right place in the right time with the strong T-cell functions, these vaccines have the potential contribute significantly to HIV-1 prevention and cure. The fact that the tHIVconsvX vaccine regions contain protective epitopes is a very encouraging first step.

## MATERIALS AND METHODS

### Subjects.

All treatment-naive Japanese subjects chronically infected with HIV-1 subtype B were recruited from the National Center for Global Health and Medicine. PBMCs were separated from whole blood. HLA types of the individuals were determined by standard sequence-based genotyping. This study was approved by the ethics committees of Kumamoto University and the National Center for Global Health and Medicine. Informed consent was obtained from all individuals according to the Declaration of Helsinki.

### Peptides.

We generated seven pools containing pairs of 15-mer Pol peptides overlapped by 11 amino acids covering two mosaic regions in the tHIVconsvX immunogen (29). Each pool contains 17 to 21 pairs of the 15-mer peptides. Pools P4, P5, P6, P7, P8, P9, and P10 cover Pol amino acids 94 to 188, 178 to 268, 258 to 352, 342 to 426, 482 to 510/741 to 798, 852 to 934, and 924 to 1003, respectively (Fig. 1). The 15-mer peptides derived from the tHIVconsvX vaccine were generously provided by the International AIDS Vaccine Initiative. Shorter mapping peptides were synthesized by utilizing an automated multiple peptide synthesizer and purified by high-performance liquid chromatography (HPLC). The purity of all peptides (>90%) was examined by HPLC and mass spectrometry.

### Cell lines.

C1R cells expressing HLA-A*26:01 (C1R-A2601), HLA-A*33:03 (C1R-A3303), HLA-B*15:01 (C1R-B1501), HLA-B*39:01 (C1R-B3901), HLA-A*31:01 (C1R-A3101), HLA-B*40:06 (C1R-B4006), or HLA-B*40:01 (C1R-B4001) were previously generated by transfecting the relevant genes into C1R cell lines (20, 42–45). 721.221 cells expressing CD4 molecules and HLA-A*02:06 (721.221-A0206), HLA-A*24:02 (721.221-A2402), HLA-A*33:03 (721.221-A3303), HLA-C*07:02 (721.221-C0702), HLA-C*08:01 (721.221-C0801), HLA-C*01:02 (721.221-C0102), HLA-C*03:04 (721.221-C0304), HLA-B*48:01 (721.221-B4801), HLA-A*02:01 (721.221-A0201), HLA-C*14:02 (721.221-C1402), HLA-C*04:01 (721.221-C0401), HLA-A*11:01 (721.221-A1101), HLA-B*40:06 (721.221-B4006), and HLA-B*51:01 (721.221-B5101) were previously generated (20, 43, 46–50). All cell lines were cultured in RPMI 1640 medium containing 10% fetal calf serum (FCS) with 0.15 mg/ml of hygromycin B.

### Expansion of HIV-1-specific T cells from HIV-1-infected individuals.

PBMCs from KI-1062, KI-1044, KI-0991, KI-1247, and KI-1249 individuals were incubated with 1 μM (1.6 to 1.9 μg/ml) 15-mer peptide pairs C256/257 or C258/259, C360/361 or C362/363, C346/347, C328/329, and C300/301, respectively, and cultured for 12 to 14 days to induce peptide-specific STCL.

### Intracellular cytokine staining (ICS) assay.

C1R and 721.221 cells prepulsed with peptide or 721.221 cells infected with HIV-1, strain NL4-3, were cocultured with STCLs in a 96-well plate for 2 h at 37°C. Brefeldin A (10 μg/ml) was then added and the cells were incubated further for 4 h. Cells were fixed with 4% paraformaldehyde and incubated in permeabilization buffer (0.1% saponin–10% FBS–phosphate-buffered saline [PBS]) after staining with allophycocyanin (APC)-labeled anti-CD8 monoclonal antibody (Mab; Dako, Glostrup, Denmark). The cells were then stained with fluorescein isothiocyanate (FITC)-labeled anti-gamma interferon (anti-IFN-γ) MAb (BD Bioscience, CA). The percentage of IFN-γ-producing cells among the CD8^+^ T-cell population was determined by FACS Canto II (BD Bioscience, CA).

### *Ex vivo* IFN-γ ELISPOT assay.

An *ex vivo* IFN-γ enzyme-linked immunospot (ELISPOT) assay was performed as previously described (20). To standardize the number of spots to spot-forming units (SFU)/10^6^ CD8^+^ T cells, we measured a frequency of CD8^+^ T cells among PBMCs using flow cytometry. Next 100,000 PBMCs from each individual were plated in each well in the ELISPOT plate that had been precoated with 5 μg/ml of anti-IFN-γ MAb 1-D1K (Mabtech, Stockholm, Sweden) at a concentration of 1 μM (1.6 to 1.8 μg/ml) of peptides. The plates were then incubated for 16 h at 37°C in 5% CO_2_, and then the cells were stained as previously described in detail (20). We calculated the number of CD8^+^ T cells plated in each well containing 100,000 PBMCs by using the frequency of CD8^+^ T cells among PBMCs and determined SFU/10^6^ CD8^+^ T cells in each well (20). The number of spots for each peptide-specific T cell response was finally calculated by subtracting the number of spots in wells without peptides. The mean + 5 standard deviations (SD) of the SFU of samples (*n* = 3) from 12 HIV-1-naive individuals for the peptide pool was 115 SFU/10^6^ CD8^+^ T cells (30). Therefore, we defined a positive IFN-γ ELISPOT response as larger than 200 SFU/10^6^ CD8^+^ T cells to exclude false-positive results.

### Establishment of T-cell lines specific for GA9, ER10, and IL-9 peptides using HLA/peptide tetramer complexes.

To establish T-cell lines specific for the GA9, ER10, and IL9 epitopes, HLA-B*40:06/GA9, HLA-A*33:03/ER10, and HLA-A*2402/IL9 tetrameric complexes (tetramers) were synthesized as previously described (51). PBMCs of HLA-B*40:06^+^ KI-1268, HLA-A*33:03^+^ KI-1427, and HLA-A*2402^+^ KI-1105 individuals were stained with phycoerythrin (PE)-conjugated specific tetramers at a concentration of 100 nM at 37°C for 30 min. The cells were then washed twice with R10, followed by staining with FITC-conjugated anti-CD8 MAb and 7-aminoactinomycin D (7-AAD) at 4°C for 30 min. The CD8^+^ T cells specific for the GA9, ER10, and IL9 epitopes were then sorted by FACS Aria (BD Bioscience, CA). The sorted cells were stimulated with 100 nM concentrations of the corresponding epitope peptides and cultured for 12 to 14 days to induce T-cell lines specific for each epitope. To confirm the purities of the specific T cells, the T-cell lines were analyzed by using the specific tetramers.

### *In vitro* virus inhibition assay.

The ability of HIV-1-specific CTLs to suppress HIV-1 replication *in vitro* was examined as previously described (33, 52). CD4^+^ T cells isolated from PBMCs of healthy donors carrying HLA-B*40:06, HLA-A*33:03, or HLA-A*24:02 were infected with HIV-1 NL4-3, and then the infected cells were cocultured with epitope-specific T-cell lines at effector/target (E:T) ratios of 1:1 and 0.1:1. On day 5 postinfection, the concentration of p24 Ag in the culture supernatant was measured by using an enzyme-linked immunosorbent assay.

### Bulk sequence of autologous virus.

Bulk sequencing of autologous plasma viral RNA from HIV-1-infected individuals was performed as described previously (53).

### Statistical analyses.

The two-tailed Mann-Whitney test was performed for comparison of two groups. Correlations between magnitudes and breadths of T-cell responses and pVL or CD4 count were statistically analyzed using Spearman rank test. *P* values of <0.05 were considered to be statistically significant.
